# Genomic Analysis of *Vulcanisaeta thermophila* Type Strain CBA1501^T^ Isolated from Solfataric Soil

**DOI:** 10.3389/fmicb.2016.01639

**Published:** 2016-10-19

**Authors:** Joon Yong Kim, Kyung June Yim, Hye Seon Song, Yeon Bee Kim, Dong-Gi Lee, Joseph Kwon, Kyung-Seo Oh, Seong Woon Roh

**Affiliations:** Biological Disaster Analysis Group, Korea Basic Science InstituteDaejeon, South Korea

**Keywords:** *Vulcanisaeta thermophila*, genome sequence, archaea, hyperthermophile, hyperthermophilic enzyme

## Introduction

Hyperthermophilic archaea have been isolated from high-temperature environments such as geothermally heated soils, sulfur-rich hot springs, and submarine volcanic habitats; optimal growth of these organisms occurs above 80°C (Stetter, 1999, 2006, 2013). The genus *Vulcanisaeta* belongs to the family *Thermoproteaceae*, order *Thermoproteales*, phylum *Crenarchaeota*, and was first proposed by Itoh et al. (2002). It currently includes 3 validly named species, that is, *Vulcanisaeta distributa* (Itoh et al., 2002), *V. souniana* (Itoh et al., 2002), and *V. thermophila* (Yim et al., 2015), as per the List of Prokaryotic Names with Standing in Nomenclature database (Parte, 2014). Members of the genus *Vulcanisaeta* are rod-shaped, anaerobic, hyperthermophilic, and acidophilic (Itoh et al., 2002). To date, 15 genomes, including two complete genomes, *V. distributa* and “*Vulcanisaeta moutnovskia*” (Mavromatis et al., 2010; Gumerov et al., 2011), have been reported for the genus *Vulcanisaeta*, as per the NCBI genome database (http://www.ncbi.nlm.nih.gov/genome/).

Hyperthermophilic enzymes are stable and active at high temperatures of >70°C (Vieille et al., 1996). These enzymes can be studied using model systems to elucidate enzyme mechanisms and evolution of proteins stable at high temperatures and to determine the higher temperature limit for enzyme stability (Vieille and Zeikus, 2001). In a previous study, *V. thermophila* CBA1501^T^ (= ATCC BAA-2415^T^ = JCM 17228^T^) was isolated from solfataric soil in the Republic of the Philippines (Yim et al., 2015). It was found to grow at 75–90°C, pH 4.0–6.0, and 0–1.0% (w/v) NaCl, with optimal growth at 85°C, pH 5.0, and 0% (w/v) NaCl. Here, a genome sequence of *V. thermophila* CBA1501^T^ has been reported and information of hyperthermophilic enzymes of high biotechnological value has been provided.

## Materials and methods

### Culture conditions and DNA extraction

In a previous study, we isolated *V. thermophila* CBA1501^T^ from the solfataric soil of the Mayon volcano in the Republic of the Philippines (Yim et al., 2015) and cultivated it on modified JCM medium no. 236 (M236) (containing per liter salt base solution: 2.94 g trisodium citrate dihydrate, 0.5 g yeast extract, 10.0 ml trace vitamins, 1.0 mg resazurin, 0.5 g Na_2_S·9H_2_O, and 20 mM thiosulfate). For DNA extraction, the strain was enriched at 80°C in M236 medium, using a serum bottle. Its genomic DNA was extracted using the G-spin total DNA extraction kit (iNtRON Biotechnology, Korea) and QuickGene DNA tissue kit S (Kurabo, Japan).

### Genome sequencing, assembly, and annotation

The genome sequences of *V. thermophila* CBA1501^T^ were sequenced at a read length of 300 bp using the Illumina MiSeq system, with paired-end library [insert size, 634–1101 bp (average 852 bp), computed by CLC Genomics Workbench 7.5.1 (CLC bio, Denmark)] constructed using the Nextera DNA Library Prep kit (illumina, USA), according to the manufacturer's instructions (Moon et al., 2015). A total of 6,939,438 reads (with 688-fold coverage) were assembled using CLC Genomics Workbench 7.5.1 with default parameters as follows: masking mode, no masking; mismatch cost, 2; insertion cost, 3; deletion cost, 3; length cost, 3; length fraction, 0.5; similarity fraction, 0.8; global alignment, no; auto-detect paired distances, yes; non-specific match handling map, randomly. To identify ribosomal RNA and transfer RNA, RNAmmer 1.2 (Lagesen et al., 2007) and tRNAscan v. 1.3.1 (Lowe and Eddy, 1997), respectively, were used. Protein coding sequences (CDSs) identification was performed using PRODIGAL v. 2.6.2 (Hyatt et al., 2012), and functional annotation was performed using EggNOG v. 4.1 (Powell et al., 2014), SEED subsystems (Overbeek et al., 2014), Swiss-Prot (UniProt, 2015), and KEGG (Kanehisa et al., 2016) databases with the USEARCH v. 8.0.1517 program (Edgar, 2010).

### Phylogenetic analysis

Similarities based on 16S rRNA gene sequences were calculated using EzBioCloud (http://www.ezbiocloud.net). Phylogenetic tree based on 16S rRNA gene sequences was constructed using MEGA5 (Tamura et al., 2011) with the neighbor-joining (Saitou and Nei, 1987), maximum-parsimony (Kluge and Farris, 1969), and maximum-likelihood (Felsenstein, 1981) methods, based on 1000 randomly generated trees.

### Comparative genomic analysis

For comparative analysis, reference genome sequences of closely related strains of the genus *Vulcanisaeta* were selected using the NCBI genome database (http://www.ncbi.nlm.nih.gov/genome/): *V. distributa* JCM 11215 (BBCT00000000), *V. distributa* DSM 14429 (CP002100), *V. distributa* JCM 11217 (BBBJ00000000), *V. souniana* JCM 11219 (BBBK00000000), *V. moutnovskia* 768-28 (CP002529), and *Vulcanisaeta* sp. strains CIS_19 (LOCG00000000), JCM 14467 (BBDM00000000), JCM 16159 (BBDN00000000), and JCM 161 (BBDO00000000). To determine the similarity between genome sequences, orthologous average nucleotide identity (OrthoANI) values of CBA1501^T^ and related strains in the genus *Vulcanisaeta* were calculated using the Orthologous Average Nucleotide Identity Tool (Lee et al., 2015), and a phylogenetic tree based on OrthoANI values was obtained using the EzBioCloud Comparative Genomics Database (EzCgDb; Chunlab; http://cg.ezbiocloud.net/). Annotated genomes of CBA1501^T^ and other related strains were subjected to homology search using the UBLAST program (Ward and Moreno-Hagelsieb, 2014) for pan-genome analysis. Then, pan-genome orthologous groups (POGs) were constructed using EzCgDb.

## Results

### General genomic features of *V. thermophila* CBA1501^T^

**Figure 1:**
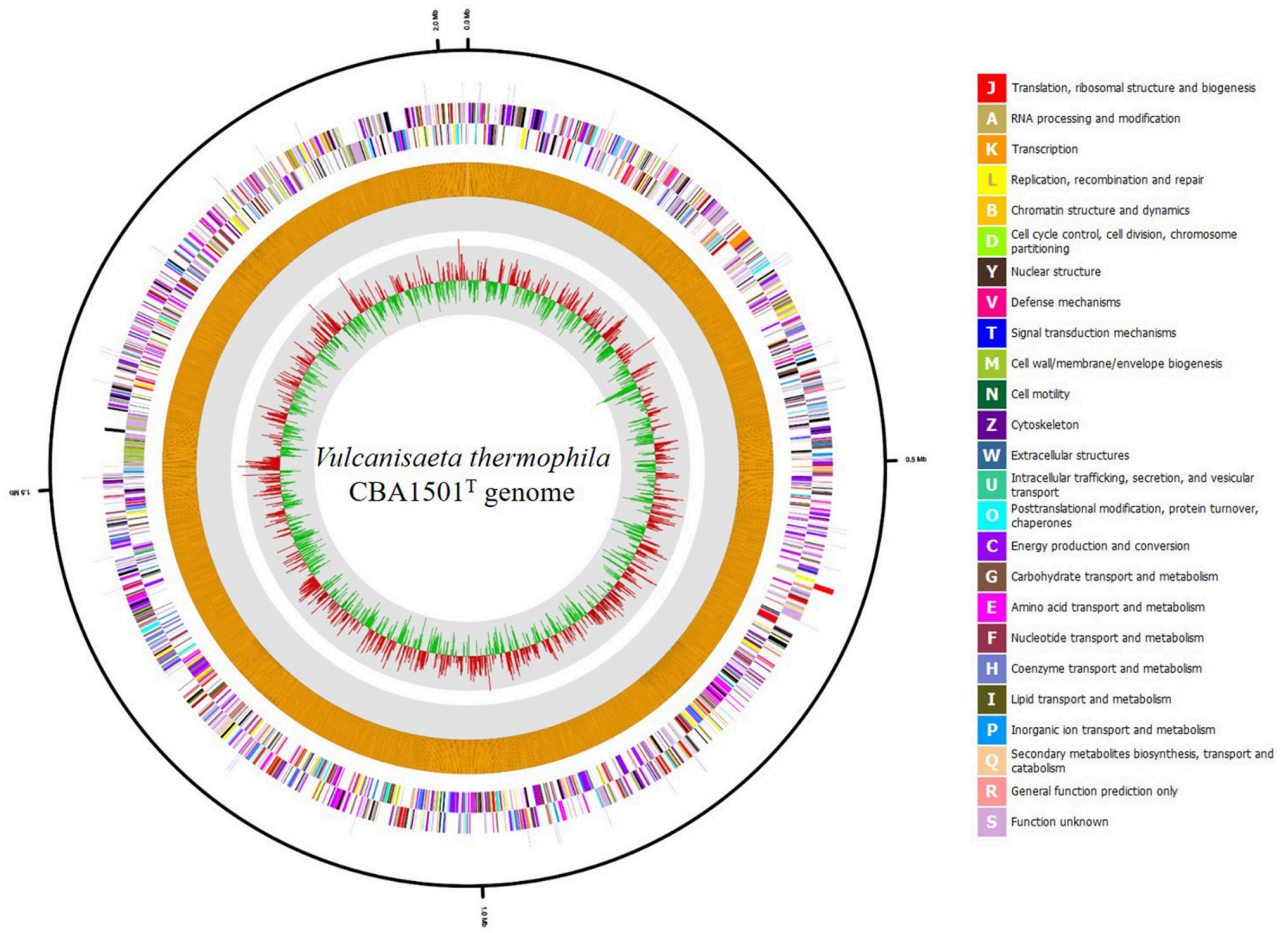
**Graphic circular map of the *Vulcanisaeta thermophila* CBA1501^T^ genome**. Outer circle shows genes on the sense and antisense strands (colored according to COG categories), and RNA genes (red, tRNA; blue, rRNA) are shown from the outside of the circle to the center. Inner circles show the GC skew, with yellow and blue indicating positive and negative values, respectively; the GC content is indicated in red and green. This genome map was visualized using CLgenomics 1.52 (Chun Lab Inc.).

The draft genome sequence of *V. thermophila* CBA1501^T^ was 2,022,594 bp in length, with a G+C content of 49.1 mol % in 10 contigs. The largest contig was 791,731 bp long, and the N50 value was 634,758 bp. The genome was found to contain 2170 CDSs, one 16S-23S-5S rRNA gene operon, and 41 tRNA genes. Genomic features are shown in Figure 1. On the basis of information from the EggNOG v. 4.1 database, 1927 genes were categorized into Clusters of Orthologous Groups of proteins (COGs) functional groups. The most abundant COG category was “Function unknown” (S; 729 genes), followed by “Energy production and conversion” (C; 178 genes), “Amino acid transport and metabolism” (E; 168 genes), “Translation, ribosomal structure and biogenesis” (J; 159 genes), “Carbohydrate transport and metabolism” (G; 96 genes), and “Coenzyme transport and metabolism” (H; 87 genes). Among the SEED subsystem categories, “Carbohydrates” (181 genes), “Amino Acids and Derivatives” (171 genes), “Protein Metabolism” (142 genes) and “Cofactors, Vitamins, Prosthetic Groups, Pigments” (116 genes) were the most dominant categories (>10% of a total of 1,094 matched SEED subsystem categories).

### Phylogenetic analysis

*V. thermophila* CBA1501^T^ had the greatest 16S rRNA gene sequence similarity with the following (in this order): *V. distributa* DSM 14429^T^ (98.6%), *Stygiolobus azoricus* DSM 6296^T^ (98.6%), *V. souniana* IC-059^T^ (97.5%), *Caldivirga maquilingensis* IC-167^T^ (94.6%), *Pyrobaculum ferrireducens* 1860^T^ (93.8%), *Pyrobaculum islandicum* DSM 4184^T^ (93.6%), *Thermoproteus uzoniensis* 768-20 (93.6%), *Pyrobaculum organotrophum* JCM 9190^T^ (93.5%), and *Thermoproteus thermophilus* CBA1502^T^ (93.2%). The phylogenetic analysis indicated that the strain CBA1501^T^ clustered with species of the genus *Vulcanisaeta* (Supplementary Figure 1A).

### Comparative genomics data

*V. thermophila* CBA1501^T^ had lesser than 73% orthoANI values with all of the related strains in the genus *Vulcanisaeta* (Supplementary Table 1). In the orthoANI values-based dendrogram, the strain CBA1501^T^ was located as an outgroup to the other related strains in *Vulcanisaeta* (Supplementary Figure 1B). These results indicate that *V. thermophila* CBA1501^T^ is evolutionarily distinct from other related strains. The pan-genome analysis showed that 10 genomes in the genus *Vulcanisaeta* have the core genome, comprised of 979 POGs. In contrast, only the genome of strain CBA1501^T^ had 211 POGs as a singleton. Among these singletons, various enzymes, including arylformamidase, shikimate kinase, formyl-CoA transferase, xanthine dehydrogenase, hydrogensulfite reductase, and amidase, were detected.

In conclusion, the information provided here is useful as the genome of *V. thermophila* CBA1501^T^ will provide insights into the metabolism of hyperthermophilic archaea and aid in identifying opportunities for biotechnological applications of novel hyperthermophilic enzymes.

## Data access

The genome sequences of *V. thermophila* CBA1501^T^ (=ATCC BAA-2415^T^ = JCM 17228^T^) were deposited in the DDBJ under the accession numbers BCLI01000001-BCLI01000010 (http://www.ncbi.nlm.nih.gov/Traces/wgs/BCLI01). The annotated data of *V. thermophila* CBA1501^T^ based on SEED subsystems is accessible on SEED viewer v. 2.0 by logging in with the guest account (Genome ID 6666666.192913, username: guest, password: guest) at the web address: http://rast.nmpdr.org/seedviewer.cgi?page=Organism&organism=6666666.192913.

## Author contributions

SWR designed and coordinated all the experiments. KJY performed cultivation, DNA extraction and purification. JYK, HSS, YBK, D-GL, JK, and K-SO performed the sequencing, genome assembly, gene prediction, gene annotation and comparative genomic analysis. JYK, KJY, and SWR wrote manuscript. All authors have read the manuscript and approved.

## Funding

This research was supported by the Basic Science Research Program through the National Research Foundation of Korea (NRF) funded by the Ministry of Education, Science, and Technology (2015R1D1A1A09061039), project fund from the Center for Analytical Research of Disaster Science of the Korea Basic Science Institute (C36703).

### Conflict of interest statement

The authors declare that the research was conducted in the absence of any commercial or financial relationships that could be construed as a potential conflict of interest.
