# Supplementary material for: Genomic Analysis of Vulcanisaeta thermophila Type Strain CBA1501T Isolated from Solfataric Soil
Source: Front Microbiol. 2016 Oct 19;7:1639. doi: 10.3389/fmicb.2016.01639 (PMC5069417; doi:10.3389/fmicb.2016.01639)
Supplement: Supplementary file 1 [file DataSheet1.docx]

Supplementary Material

Genomic Analysis of Vulcanisaeta thermophila Type Strain CBA1501^T^ Isolated from Solfataric Soil

Joon Yong Kim, Kyung June Yim, Hye Seon Song, Yeon Bee Kim, Dong-Gi Lee, Joseph Kwon Kyung-Seo Oh, Seong Woon Roh^*^

*** Correspondence:**

Seong Woon Roh:

seong18@gmail.com

**1. Supplementary Figures and Tables**

**1.1 Supplementary Figures**

**
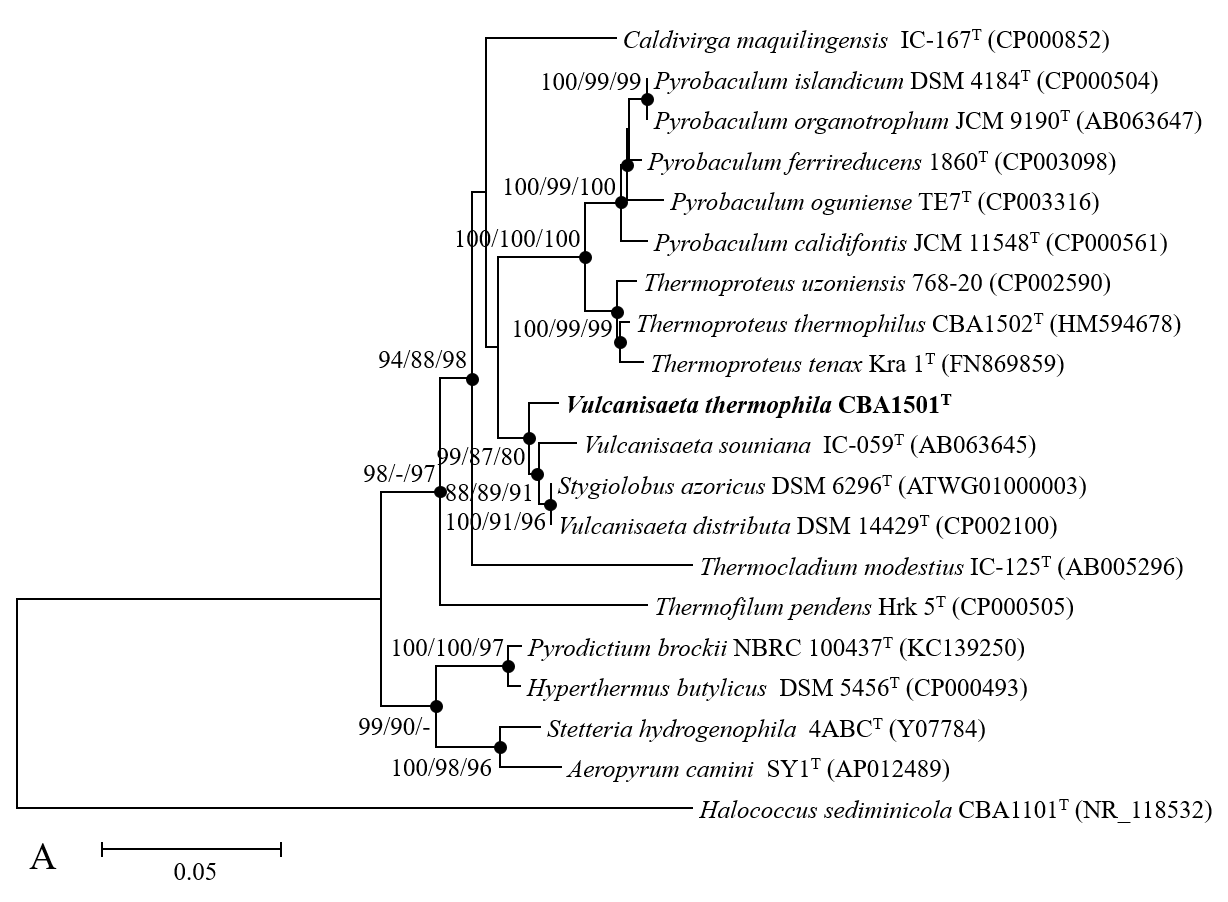

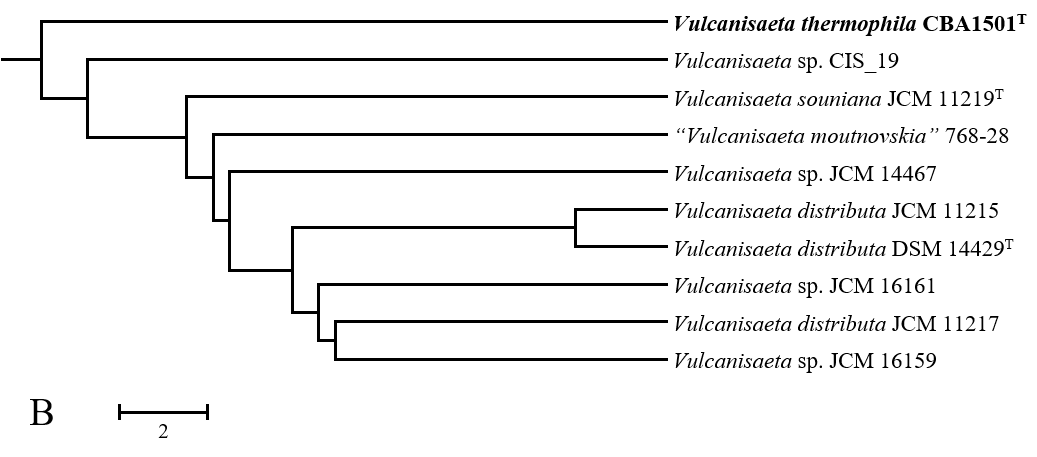
**

**Supplementary Figure 1.** Phylogenetic consensus tree based on 16S rRNA gene sequence and orthoANI values. (A) Neighbour-joining (NJ) algorithm for the 16S rRNA gene sequence showing the taxonomic position of strain CBA1501^T^. The closed circles indicate the nodes were identified using both the maximum-parsimony (MP) and maximum-likelihood (ME) algorithms. The numbers at nodes represent bootstrap values (>70%), were calculated using NJ/MP/ME probabilities based on 1000 replicates. *Halococcus sediminicola* CBA1101^T^ was used as an outgroup. Bar, 0.05 accumulated changes per nucleotide; (B) ANI phylogenetic tree. Using EzCgDb, phylogenetic tree was constructed based on orthoANI values.

**1.2 Supplementary Tables**

**Supplementary Table 1.** OrthoANI values between *Vulcanisaeta thermophila* CBA1501^T^ and related strains.

|  | *Vulcanisaeta distributa*  JCM 11215 | | *Vulcanisaeta sp.*  CIS_19 | *Vulcanisaeta distributa*  DSM 14429^T^ | "*Vulcanisaeta moutnovskia*" 768-28 | *Vulcanisaeta distributa* JCM 11217 | *Vulcanisaeta souniana*  JCM 11219^T^ | *Vulcanisaeta* sp.  JCM 14467 | *Vulcanisaeta* sp.  JCM 16159 | *Vulcanisaeta* sp.  JCM 16161 | ***Vulcanisaeta thermophila* CBA1501^T^** |
| --- | --- | --- | --- | --- | --- | --- | --- | --- | --- | --- | --- |
| *V. distributa*  JCM 11215 | | - | 73.8814 | 95.8885 | 79.0184 | 84.6378 | 80.0373 | 80.1042 | 81.972 | 82.4735 | 71.7746 |
| *V. sp.*  CIS_19 | - | | - | 74.1103 | 74.0086 | 74.1392 | 72.9991 | 74.1035 | 74.0348 | 74.2867 | 71.9203 |
| *V. distributa*  DSM 14429^T^ | - | | - | - | 79.7173 | 84.6478 | 80.2552 | 80.7691 | 82.4382 | 82.9135 | 72.0439 |
| "*V. moutnovskia*"  768-28 | *-* | | - | - | - | 80.0844 | 78.1484 | 78.3411 | 79.945 | 80.484 | 71.1368 |
| *V. distributa*  JCM 11217 | - | | - | - | - | - | 77.798 | 80.4878 | 84.9694 | 84.629 | 71.6267 |
| *V. souniana*  JCM 11219^T^ | - | | - | - | - | - | - | 76.6858 | 78.2638 | 77.4468 | 71.329 |
| *V.* sp.  JCM 14467 | - | | - | - | - | - | - | - | 79.6766 | 80.5415 | 72.418 |
| *V.* sp.  JCM 16159 | - | | - | - | - | - | - | - | - | 84.0625 | 72.2048 |
| *V.* sp.  JCM 16161 | - | | - | - | - | - | - | - | - | - | 72.0858 |
| ***Vulcanisaeta. thermophila***  **CBA1501^T^** | - | | - | - | - | - | - | - | - | - | - |
